# Severe Melancholia Consequent upon Pelvic Disease

**Published:** 1901-06

**Authors:** Chas. Wilson

**Affiliations:** Interne.


### Clinical Reports.

### I.—SEVERE MELANCHOLIA CONSEQUENT UPON PELVIC DISEASE.

By L. G. HANLEY, M. D.,
Gynecologist, Buffalo Hospital of the Sisters of Charity.
Reported by CHAS. WILSON, M. D., Interne.

MRS. B., aged 30, married, was committed to Retreat for Insane, October 30, 1900. Nativity of parents not known; habits, temperate. Was treated for nervous prostration a year and a half before admission. At time of entrance was talkative, excited, and, again, at times quite depressed. Husband stated that she had lost all interest in her household duties, and he frequently found her sitting for hours at a time brooding and quite melancholic. She was treated for two years by her family physician, who advised she be sent to an institution for the cure of the insane. While under treatment at the institution, it was discovered that she was suffering from pelvic disease, and was referred to Doctor Hanley, at the Sisters' of Charity Hospital, December 10, 1900.

History and examination shows that she has had three children, last child born in 1897; labors, hard, and forceps used in each case. Menstruation began at 14 years of age; regular—very little, pale. Patient also has exophthalmic goitre. Is very melancholic, sometimes refuses to answer when spoken to, and requires constant watching. Complains of pain in lower abdomen, and says she feels worse after treatment. She says that treatments make her worse, and has had pain for four years. During that time she says her doctor treated her for womb trouble, but always felt that she could stand no more treatments, as she expressed it—"they always made her feel worse."

Pelvic examination shows a bilateral laceration of cervix markedly inflamed, with enlargement of both ovaries and tubes, with a complete retroversion. Patient prepared for celiotomy and trachelorraphy. When abdomen was opened, each ovary was found to be about four times its normal size, prolapsed, in a state of cystic degeneration with marked adhesions. Both tubes and ovaries removed; also laceration of cervix repaired. Patient sat up on the twentieth day; union of cervix and abdominal wound perfect. During her stay at the hospital she showed no evidences of mental aberration after the first week. She continued to improve mentally, seemed anxious to return to her home and expressed a desire to be with her husband and her family.

She was discharged January 27, 1901. Information received March 20th from her shows that she is feeling very well, has gained in weight, and says she feels perfectly happy and contented. Evidently this is conclusive proof that her mental psychosis was consequent upon her pelvic disease.

